# Refinement of the karyological aspects of *Psidium guineense* (Swartz, 1788): a comparison with *Psidium guajava* (Linnaeus, 1753)

**DOI:** 10.3897/CompCytogen.v10i1.6462

**Published:** 2016-02-02

**Authors:** Anelise Machado Marques, Amélia Carlos Tuler, Carlos Roberto Carvalho, Tatiana Tavares Carrijo, Marcia Flores da Silva Ferreira, Wellington Ronildo Clarindo

**Affiliations:** 1 Laboratório de Citogenética, Departamento de Biologia, Centro de Ciências Agrárias, Universidade Federal do Espírito Santo, CEP: 29.500-000 Alegre – ES, Brazil; 2 Escola Nacional de Botânica Tropical, Instituto de Pesquisas Jardim Botânico do Rio de Janeiro, CEP: 22.460-036 Rio de Janeiro – RJ, Brazil; 3 Laboratório de Citogenética e Citometria, Departamento de Biologia Geral, Centro de Ciências Biológicas e da Saúde, Universidade Federal de Viçosa, CEP: 36.570-000 Viçosa – MG, Brazil; 4 Laboratório de Botânica, Departamento de Biologia, Centro de Ciências Agrárias, Universidade Federal do Espírito Santo, CEP: 29.500-000 Alegre – ES, Brazil; 5 Laboratório de Genética e Melhoramento Vegetal, Departamento de Biologia, Centro de Ciências Agrárias, Universidade Federal do Espírito Santo, CEP: 29.500-000 Alegre – ES, Brazil

**Keywords:** Psidium, polyploidy, karyotype evolution, cytogenetic, flow cytometry, SSR markers

## Abstract

Euploidy plays an important role in the evolution and diversification of *Psidium* Linnaeus, 1753. However, few data about the nuclear DNA content, chromosome characterization (morphometry and class) and molecular markers have been reported for this genus. In this context, the present study aims to shed light on the genome of *Psidium guineense* Swartz, 1788, comparing it with *Psidium guajava* Linnaeus, 1753. Using flow cytometry, the nuclear 2C value of *Psidium guineense* was 2C = 1.85 picograms (pg), and the karyotype showed 2n = 4x = 44 chromosomes. Thus, *Psidium guineense* has four chromosome sets, in accordance with the basic chromosome number of *Psidium* (x = 11). In addition, karyomorphometric analysis revealed morphologically identical chromosome groups in the karyotype of *Psidium guineense*. The high transferability of microsatellites (98.6%) further corroborates with phylogenetic relationship between *Psidium guajava* and *Psidium guineense*. Based on the data regarding nuclear genome size, karyotype morphometry and molecular markers of *Psidium guineense* and *Psidium guajava* (2C = 0.95 pg, 2n = 2x = 22 chromosomes), *Psidium guineense* is a tetraploid species. These data reveal the role of euploidy in the diversification of the genus *Psidium*.

## Introduction

*Psidium* Linnaeus, 1753 is a genus of Myrtaceae that comprises about 92 species (Govaerts et al. 2013), predominantly distributed in the Neotropics. The species of this genus differ from those belonging to other Myrtaceae genera by seeds with bony testa, cochlear embryo with small cotyledons and large hypocotyl (Landrum and Kawasaki 1997). Brazil is a relevant center of *Psidium* species diversity, comprising approximately 60 taxa widely distributed in different biomes (Sobral et al. 2014). The genus is economically important (Rai et al. 2010), with *Psidium guajava* Linnaeus, 1753, *Psidium cattleyanum* Sabine, 1821 and *Psidium guineense* Swartz, 1788 being the most relevant commercial species for fruit production and/or source of compounds in the pharmaceutical industry. Of these taxa, *Psidium cattleianum* (Costa and Forni-Martins 2007, Costa et al. 2008, Souza et al. 2015) and *Psidium guajava* (Costa and Forni-Martins 2007, Coser et al. 2012) are the best-known species with regard to cytogenetic features.

Karyotypic characterization has been applied to better understand the changes that occur during genome evolution (Éder-Silva et al. 2007). Based on previous cytogenetic studies, euploidy has led to diversification in *Psidium* (Briggs and Walters 1997). In fact, a series of euploid organisms, such as diploid (2n = 22), tetraploid (2n = 44), hexaploid (2n = 66) and octoploid (2n = 88) species (Atchison 1947, Costa and Forni-Martins 2006a, 2006b, 2007), derived from the basic x = 11 chromosome number (Atchison 1947, Costa et al. 2008), has been reported for the genus. Nevertheless, the relationship among species that arose from euploidy events is still poorly understood in *Psidium*.

According to current knowledge, few *Psidium* species are diploid (2n = 22), such as *Psidium chinense* Loudon, 1830 (Naitani and Srivastava 1965), *Psidium friedrichsthalianum* Niedenzu, 1893 and *Psidium guajava*, which is the only diploid species whose karyotype has been characterized (Coser et al. 2012). Considering that the genus *Psidium* shows polyploid species (2n = 44–88 chromosomes), the allo- and/or autopolyploidization in diploid species of this genus can be related to the occurrence of polyploidy. Thus, the chromosome number and karyotype characterization of the polyploid species represents the basis to understand the origin and diversification in *Psidium*.

Euploid species are key models for evolution because they provide evidence of the polyploidization event that promoted diversification and speciation. Considering that, this study aimed to refine the knowledge about karyological aspects of *Psidium guineense*. Besides, a comparison was performed with the diploid species (2x = 22) *Psidium guajava*, because this species is the only of the *Psidium* genus characterized from flow cytometry (FCM), cytogenetic (Coser et al. 2012) and molecular markers (Risterucci et al. 2005, Guavamap 2008, Nogueira et al. 2015).

## Material and methods

*Psidium guajava* fruits were obtained from 50 plants growing in orchards located in different regions of the Brazil. *Psidium guineense* fruits were obtained from indigenous populations occurring in Atlantic Forest remnants located in the Municipalities of Alegre (four individuals), Itapemirim (three individuals), Santa Teresa (seven individuals), and Conceição da Barra (six individuals), all located in Espírito Santo state. The sampling was done between 2012 and 2014.

FCM and molecular analyses were conducted with the same 50 individuals of *Psidium guajava* and 20 of *Psidium guineense*. Due to FCM results, karyotype characterization was performed using seeds obtained from ten distinct plants of the two species. *Solanum lycopersicum* Linnaeus, 1753, ‘Stupické’ (reference standard for FCM, 2C = 2.00 picograms – pg; Praça-Fontes et al. 2011) seeds were supplied by Dr. Jaroslav Doležel (Experimental Institute of Botany – Czech Republic).

### 2C nuclear measurement

Leaves were collected from *Solanum lycopersicum* (standard), *Psidium guajava* and *Psidium guineense* (samples). Nuclei suspensions were obtained from leaf fragments of the standard and of each sample, according to a previously described protocol (Otto 1990, Coser et al. 2012). These suspensions were analyzed in a Partec PAS® flow cytometer (Partec® GmbH, Munster – Germany) equipped with a laser source (488 nm). Nuclei-emitted propidium iodide fluorescence was collected by an RG 610-nm band-pass filter. The equipment was calibrated for linearity and aligned with microbeads and standard solutions according to the manufacturer’s recommendations. FloMax® software (Partec®) was used for the data analysis. Six independent replicates were performed for each individual, with over 10,000 nuclei analyzed per replicate. The mean 2C values of *Psidium guajava* and *Psidium guineense* were calculated by dividing the mean channel of the G_0_/G_1_ fluorescence peak for the reference standard by the mean channel of the G_0_/G_1_ peak for each sample.

### Karyotype characterization

Seeds of *Psidium guineense* and *Psidium guajava* were germinated in Petri dishes containing distilled water (dH_2_O) at 30 °C. The roots showing 1.0–2.0 cm in length were treated for a period of 4, 15 or 19 h with the microtubule-inhibiting agents amiprophos-methyl (APM, Nihon Bayer Agrochem K. K.®) or oryzalin (ORY, Sigma®) at a final concentration of 4 µM. Subsequently, the roots were washed with dH_2_O for 20 min, then fixed in fresh methanol:acetic acid (Merck®) solution (3:1). The fixative was changed three times, and the roots were stored at -20 °C for 24 h. The roots were washed and incubated for 2:00, 2:15 or 2:30 h at 34 °C in pectinase solution (Sigma®, E6287) at ratios of 1:8, 1:10, 1:12 or 1:15 (enzyme:water). Next, the roots were washed for 10 min in dH_2_O, fixed once more, and stored at -20 °C (Coser et al. 2012). Slides were prepared using the techniques of root meristem dissociation and air-drying (Carvalho et al. 2007). The slides were analyzed and the chromosome images were captured with a Media Cybernetics® Evolution^TM^
charge-coupled device (CCD) video camera mounted on a Nikon 80i microscope (Nikon – Japan).

### Molecular analysis

The genomic DNA was extracted from young leaves according to Doyle and Doyle (1990). The integrity and concentration of the DNA samples were verified using a Nanodrop^TM^ 2000. Amplification reactions were performed using 142 simple sequence repeat (SSR) markers (Suppl. material 1) designed for *Psidium guajava* (Risterucci et al. 2005, Guavamap 2008). Each amplification reaction consisted of 15 µL of solution containing: 60 ng DNA, 0.3 µM of each primer, 1.5 U Taq polymerase DNA (Phoneutria), 1.7 µM MgCl_2_ and 0.2 µM dNTPs. The following program was used: denaturation at 94 °C for 4 min, followed by 35 cycles of denaturation at 94 °C for 45 s, annealing at temperature (T_a_) of 50 °C or 55 °C for 1 min, and extension at 72 °C for 8 min. The reactions were performed in a Veriti® 96-Well Thermal Cycler ABI. The amplification products were separated using 6% polyacrylamide gel electrophoresis, stained with ethidium bromide, and photographed using a photo-documentation system (ChemiDoc XRS + System – Bio-Rad^TM^). For confirmation, up to three independent replications were performed.

## Results and discussion

The FCM protocol, using isolation buffer for 10 min and staining buffer for 30 min, provided peaks relative to G_0_/G_1_ nuclei with coefficient of variation (CV) lower than 3.46%, and thus high resolution. This result indicates that the suspensions contained sufficient number of intact, isolated and stoichiometrically stained nuclei.

**Figure 1.**
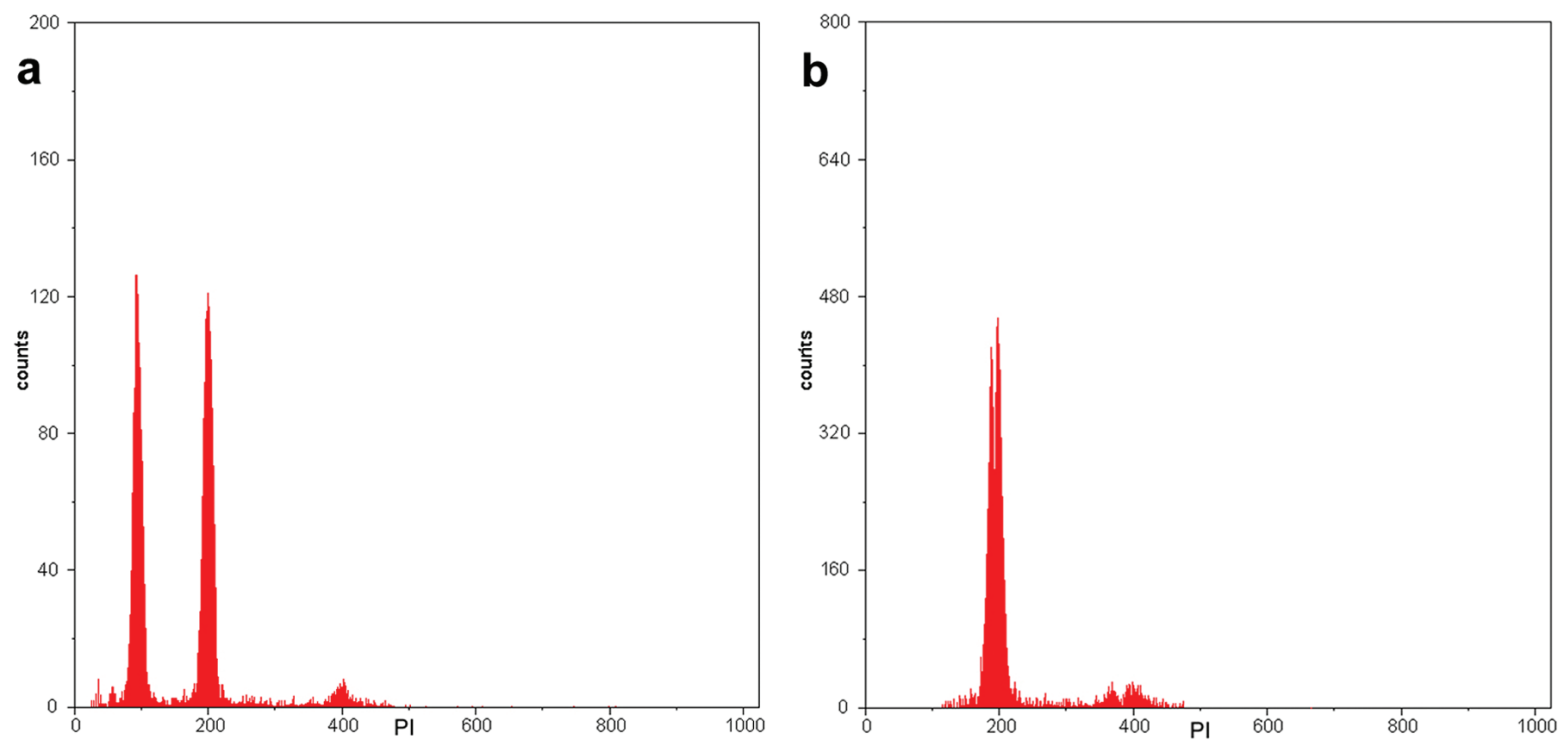
Representative histograms obtained from FCM analysis of nuclear suspensions stained with propidium iodide. **A** G_0_/G_1_ nuclei peak of the sample *Psidium guajava* (2C = 0.95 pg), positioned in channel 95, and the standard *Solanum lycopersicum* (2C = 2.00 pg) in channel 200 **B** G_0_/G_1_ nuclei peak of the sample *Psidium guineense* (2C = 1.85 pg), positioned in channel 185, and the standard *Solanum lycopersicum* (2C = 2.00 pg) in channel 200.

Based upon the large number of plant samples of distinct genotypes evaluated in this study, the mean nuclear 2C value is 0.95 pg for all *Psidium guajava* plants (Fig. 1a) and 1.85 pg for all *Psidium guineense* pants (Fig. 1b). The 2C values of *Psidium guajava* and *Psidium guineense* are small compared with those of most angiosperms, according to reference values defined by Bennett and Leitch (2011). Similarly, low 2C DNA content values were also found in some Myrtaceae, such as the genus *Eucalyptus* L’Hér. 1789, which varies from 0.80 to 1.50 pg.

*Psidium guajava* was one of the first Myrtaceae species for which the nuclear genome size was measured using Feulgen microdensitometry. With this method, mean values of 2C = 0.66 pg (Bennett and Smith 1976) and 2C = 1.24 pg (Ohri 2002) were obtained. Nuclear DNA content has also been measured for *Psidium guajava* using FCM, and the mean values were 2C = 0.507 pg (‘White’), 2C = 0.551 pg (‘Red’, Costa et al. 2008), 2C = 0.95 pg (28 genotypes, Coser et al. 2012), 2C = 0.99 pg (‘Paluma’) and 2C = 1.02 pg (‘Purple’, Souza et al. 2015). In the present study, the 2C value for *Psidium guineense* was 2C = 1.85 pg, approximately twice that observed in *Psidium guajava* (2C = 0.95 pg). The 2C value of *Psidium guineense* has also been measured as 2C = 2.02 pg (Souza et al. 2015).

The distinct 2C values observed for *Psidium guineense* and *Psidium guajava* may be related to the different techniques, plant standards, nuclear isolation and staining procedures used. More inconsistent values of DNA content were found by Costa et al. (2008), who used *Arabidopsis thaliana* Linnaeus, 1753, ‘Columbia’ (2C = 0.32 pg) as reference standard. The leaf of this species exhibits endopolyploidy (2C, 4C, 8C…) (Yotoko et al. 2011); thus, it is necessary to correctly check the reference G_0_/G_1_ peak to measure the 2C value of the sample based on the 2C nuclei of this standard.

**Figure 2.**
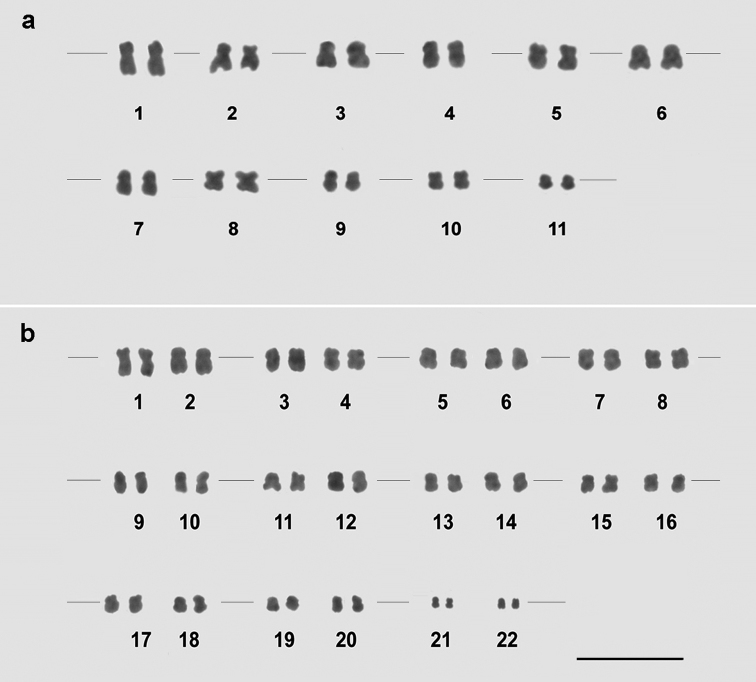
**A** *Psidium guajava* karyogram showing 2n = 2x = 22 chromosomes, being five metacentric (3, 4, 8, 9, 10) and six submetacentric pairs (1, 2, 5, 6, 7, 11) **B** *Psidium guineense* karyogram showing 2n = 4x = 44 chromosomes, with two metacentric (11, 12) and twenty submetacentric pairs (1–10, 13–22). Note groups of morphologically identical chromosomes, such as 1 and 2, 3 and 4, 21 and 22. Bar = 5 μm.

Based on DNA content, the occurrence of karyotype modifications that increased the genome size may have played a role in the origin of *Psidium guineense*. To confirm this hypothesis, karyotypic characterization was accomplished for *Psidium guineense* and *Psidium guajava*. The root tips that were treated with 4 µM APM for 15 h and macerated in 1:10 pectinase solution for 2 h provided the most adequate metaphases for morphometric analysis. Metaphases were chosen based on relevant characteristics: well-spread chromosomes with well-defined constriction, without chromatin deformations and cytoplasmic background noise. These features allowed accurate chromosome counting, morphometric characterization and assembly of the karyograms (Fig. 2, Table 1).

**Table 1.** Morphometric data and classification of the chromosomes of *Psidium guajava* and *Psidium guineense*. The mean values of total length, short and long arms were measured using at least 10 metaphases of each species.

*Psidium guajava*						*Psidium guineense*					
Chrom^a^	Total^b^	Short arm^b^	Long arm^b^	r^c^	Class^d^	Chrom^a^	Total^b^	Short arm^b^	Long arm^b^	r^c^	Class^d^
1	2.03	0.73	1.30	1.78	SM	1–2	1.82	0.63	1.20	1.91	SM
2	1.60	0.52	1.08	2.08	SM	3–4	1.60	0.60	1.00	1.67	SM
3	1.57	0.64	0.93	1.45	M	5–6	1.53	0.57	0.96	1.70	SM
4	1.52	0.73	0.79	1.08	M	7–8	1.44	0.55	0.89	1.62	SM
5	1.47	0.58	0.89	1.53	SM	9–10	1.30	0.50	0.80	1.60	SM
6	1.42	0.53	0.89	1.68	SM	11–12	1.25	0.58	0.68	1.17	M
7	1.37	0.53	0.84	1.58	SM	13–14	1.20	0.48	0.73	1.53	SM
8	1.15	0.56	0.59	1.05	M	15–16	1.17	0.43	0.74	1.70	SM
9	1.12	0.45	0.67	1.49	M	17–18	1.06	0.30	0.76	2.53	SM
10	1.05	0.50	0.55	1.10	M	19–20	0.93	0.25	0.68	2.73	SM
11	0.85	0.27	0.58	2.15	SM	21–22	0.66	0.20	0.46	2.29	SM
Total^e^	15.15						13.96				

^a^Chrom – chromosome of *Psidium guajava* and chromosome groups of *Psidium guineense*; ^b^Length in µm; ^c^Measured by arm ratio – long/short; ^d^Class: M – metacentric and SM – submetacentric; ^e^Total value based on basic chromosome number X = 11.

The chromosome number of *Psidium guajava* and of *Psidium guineense* were accurately determined here as 2n = 2x = 22 and 2n = 4x = 44, respectively (Fig. 2, Table 1). Thus, no intraspecific karyotype variations were identified for all *Psidium guajava* and *Psidium guineense* plants. Differently, other studies have reported cytotypes for *Psidium guineense* (Srivastava 1977) and mainly for *Psidium guajava* (Kumar and Ranade 1952, Majumder and Mukherjee 1972, Srivastava 1977, Costa and Forni-Martins 2006a, Éder-Silva et al. 2007, Souza et al. 2015), indicating the occurrence of an intraspecific chromosome variation related to euploidy and aneuploidy. During all period of the experiments (2012–2014), none plant exhibiting somatic chromosome number variation was recorded for both *Psidium* species.

*Psidium guajava* exhibited metacentric (pairs 3, 4, 8, 9, 10) and submetacentric chromosomes (pairs 1, 2, 5, 6, 7, 11). This species had relatively small and morphologically similar chromosomes, two of which (1 and 11) were distinguished by their very distinct total length. Paredes et al. (2006) reported variation in the morphometric classification of the chromosomes of some *Psidium guajava* genotypes, relating seven metacentric, two submetacentric and two acrocentric chromosome pairs. However, the same authors reported eight metacentric, one submetacentric and two acrocentric chromosome pairs in other genotypes. Coser et al. (2012) studied for the first time the morphometric characterization of *Psidium guajava* using enzymatic cellular dissociation of the roots and air-drying of the slides. The authors observed that, independently of genotype, *Psidium guajava* has 2n = 2x = 22 chromosomes with five metacentric (3, 4, 8, 9, 10) and six submetacentric pairs (1, 2, 5, 6, 7, 11).

As observed for *Psidium guajava*, the karyotype of *Psidium guineense* also showed only metacentric (11, 12) and submetacentric (1–10, 13–22) chromosomes (Table 1). Previous cytogenetic approaches revealed a karyotype for *Psidium guineense* of 2n = 4x = 44 chromosomes (Chakraborti et al. 2010). Besides metacentric and submetacentric chromosomes, Chakraborti et al. (2010) also reported an acrocentric one, as well as a chromosome pair distinguished by a secondary constriction for *Psidium guineense*. The two latter features were not found in the present work.

The karyomorphometric analysis also revealed groups of morphologically identical chromosomes in *Psidium guineense*: 1–2, 3–4, 5–6, 7–8, 9–10, 11–12, 13–14, 15–16, 17–18, 19–20 and 21–22 (Table 1). Therefore, the cytogenetic procedures discriminated 11 chromosome groups, equivalent to the basic chromosome number of the genus *Psidium*. Based on total size and class, the previous study performed by Chakraborti et al. (2010) identified only four chromosome groups (A, B, C and D) for *Psidium guineense*.

Considering the basic chromosome number of *Psidium* (x = 11) (Atchison 1947, Costa et al. 2008), the cytogenetic data suggest the origin of *Psidium guineense* from a polyploidization event. Therefore, the cytogenetic data confirm the FCM results in which the mean DNA contents of *Psidium guajava* (2C = 0.95 pg) and *Psidium guineense* (2C = 1.85 pg) indicate the polyploidy origin of the latter species. Polyploid species have been reported for *Psidium* (Atchison 1947, Andrade and Forni-Martins 1998, Costa and Forni-Martins 2006a, 2006b, 2007, Costa et al. 2008), as tetraploid (2n = 44, *Psidium acutangulum* Candolle, 1828, *Psidium cattleyanum* Sabine, *Psidium grandifolium* Candolle, 1828, *Psidium friedrichsthalianum* and *Psidium guineense*), hexaploid (2n = 66, *Psidium cattleyanum*) and octoploid (2n = 88, *Psidium cattleyanum*) plants.

From meiotic analysis in *Psidium guineense*, Chakraborti et al. (2010) related the occurrence of 22 bivalents and, consequently, of a Mendelian segregation in anaphases. These facts and cytogenetic data found here suggest that *Psidium guineense* is a true allopolyploid. A true allopolyploid is a hybrid formed through reproductive cells of species with different karyotypes (Stebbins 1947). Due of this, homologous chromosomes paring in meiosis, enabling the establishment only of bivalents and the formation of viable reproductive cells. Therefore, the reproductive behavior of the true allopolyploids is like a diploid species, allowing the maintenance of the ploidy level during the generations, as observed for *Psidium guineense* (Chakraborti et al. 2010).

The variation in chromosome number seen in the genus *Psidium* can promote genetic isolation and possibly create barriers to gene flow (Stace 1991), leading to speciation (Briggs and Walters 1997). Polyploidy is considered one of the main mechanisms of evolution in plants (Soltis et al. 2003). Auto- or allopolyploids may exhibit genetic and phenotypic alterations compared with their ancestral species (Soltis and Soltis 1999, Mable 2003). These changes can be observed in the first generation after polyploidization or hybridization, and also along the evolutionary history of the polyploid, leading to increased diversity (Soltis and Soltis 1999, Soltis et al. 2009, Weiss-Schneeweiss et al. 2013).

Among the 142 SSR markers, 140 were amplified in *Psidium guineense*, representing 98.6% of transferability. The high amplification rate (98.6%) found for the *Psidium guajava* SSR primers in *Psidium guineense* showed that the annealing regions are conserved in both species, revealing the high similarity between them. This result also evidenced that these DNA sequences of *Psidium guineense* are very similar in relation to *Psidium guajava*, since values of cross-amplification of approximately 73% have been reported for species of the same genus (Barbará et al. 2007). According to Barbará et al. (2007) and Nogueira et al. (2015), the transferability rate of the SSR is higher among species phylogenetically related due to conservation of the sequences between them. Due this fact, SSR markers have been used to compare the similarity level between the genome of distinct species, allowing to analyze the phylogenetic relationship (Buschiazzo and Gemmell 2010, Meglécz et al. 2012, Nogueira et al. 2015). As well as for SSR markers, *Psidium guajava* and *Psidium guineense* exhibit strong morphological similarity between them. This fact makes it laborious to identify these species at specific level. Based on this fact, in this study, *Psidium guajava* and *Psidium guineense* were distinguished from leaf (number of veins, hairiness scattered over the abaxial leaf and adaxial) and floral (apiculus) structures.

Of the 140 primers, 117 were chosen to determine the total number of alleles, which varied from 170 for *Psidium guineense* to 148 for *Psidium guajava* (Suppl. material 1). The occurrence of three and four alleles in *Psidium guineense* for 9.6% of the primers in comparison to 3.4% in *Psidium guajava* (2x = 22) corroborates the polyploid origin of *Psidium guineense* (4x = 44) evidenced by nuclear DNA content and karyotype. Besides that, the molecular data reveal the occurrence of some duplicated sequences, such as the 316 and 422 SSR loci (Suppl. material 1), which showed three allele forms in both species. Based on these results, SSR markers can be considered an important complementary tool to study the genome evolution in *Psidium*, as is already the case for investigating the genome of vertebrates (Buschiazzo and Gemmell 2010).

This study points to the tetraploidy origin of *Psidium guineense*. These results reveal the importance of combining cytogenetic and molecular markers for a better understanding of how euploid events have influenced the speciation process in angiosperms.

## Supplementary materials

Supplementary material 1
SSR locus, annealing temperature (TA), motif, and allele number amplified in the genome of *Psidium guineense* and *Psidium guajava*. Data type: Word document. Explanation note: The Supplementary material 1 summarizes the SSR locus, annealing temperature (TA), motif, and allele number amplified in the genome of *Psidium guineense* and *Psidium guajava*. This dataset is made available under the Open Database License (http://opendatacommons.org/licenses/odbl/1.0/). The Open Database License (ODbL) is a license agreement intended to allow users to freely share, modify, and use this Dataset while maintaining this same freedom for others, provided that the original source and author(s) are credited.Anelise Machado Marques, Amélia Carlos Tuler, Carlos Roberto Carvalho, Tatiana Tavares Carrijo, Marcia Flores da Silva Ferreira, Wellington Ronildo Clarindo
