# Supplementary material for: Refinement of the karyological aspects of Psidium guineense (Swartz, 1788): a comparison with Psidium guajava (Linnaeus, 1753)
Source: Comp Cytogenet. 2016 Feb 2;10(1):117–28. doi: 10.3897/CompCytogen.v10i1.6462 (PMC4856930; doi:10.3897/CompCytogen.v10i1.6462)
Supplement: Supplementary material 1 — SSR locus, annealing temperature (TA), motif, and allele number amplified in the genome of Psidium guineense and Psidium guajava. [file CompCytogen-010-117-s001.doc]

**Appendix 1.** SSR locus, annealing temperature (T_A_), motif, and allele number amplified in the genome of *P. guineense* and *P. guajava*.

| **SSR locus** | **T_A_** | **Motif** | **Allele number for each SSR primer in *P.guineense*** | **Allele number for each SSR primer in *P. guajava*** |
| --- | --- | --- | --- | --- |
| mPgCIR 1 | 55 | (GA)_17_ | 1 | 2 |
| mPgCIR 2 | 55 | (GA)_20_ | 2 | 1 |
| mPgCIR 3 | 55 | (GA)_40_ | 1 | 1 |
| mPgCIR 9 | 55 | (GA)_19_ | 2 | 1 |
| mPgCIR 10 | 55 | (CT)_12_ | 2 | 1 |
| mPgCIR 11 | 55 | (CT)_17_ | 2 | 1 |
| mPgCIR 13 | 55 | (AC)_12_(AT)_4_G(GA)_2_ | 2 | 2 |
| mPgCIR 14 | 55 | (GA)_11_ | 2 | 1 |
| mPgCIR 15 | 55 | (GA)_8_GG(GA)_9_ | 2 | 1 |
| mPgCIR 16 | 55 | (TC)_25_ | 1 | 2 |
| mPgCIR 17 | 55 | (CT)_23_ | 1 | 1 |
| mPgCIR 18 | 55 | (GA)_23_ | 1 | 1 |
| mPgCIR 19 | 55 | (CT)_16_ | 1 | 1 |
| mPgCIR 20 | 55 | (CT)_14_(CA)_17_ | 2 | 1 |
| mPgCIR 23 | 55 | (TA)_4_(GT)_7_ | 1 | 1 |
| mPgCIR 25 | 55 | (GA)_24_ | 1 | 1 |
| mPgCIR 26 | 55 | (GT)_2_(GA)_17_ | 4 | 2 |
| mPgCIR 29 | 50 | (GA)_8_ / (GAA)_5_ | 1 | 1 |
| mPgCIR 30 | 55 | (GA)_30_ | 1 | 1 |
| mPgCIR 31 | 50 | (GA)_24_ | 1 | 1 |
| mPgCIR 34 | 50 | (GA)_16_ | 1 | 1 |
| mPgCIR 39 | 50 | (GA)_17_ | 1 | 1 |
| mPgCIR 40 | 50 | (GTA)_6_ | 1 | 3 |
| mPgCIR 46 | 56 | (GA)_36_ | 2 | 1 |
| mPgCIR 91 | 56 | (GA)_16_/(GGGA)_3_ | 1 | 2 |
| mPgCIR 94 | 50 | (GA)_18_/(GT)_6_ | 1 | 1 |
| mPgCIR 97 | 50 | (GGAA)_3_/(GGTC)_3_ | 2 | 1 |
| mPgCIR 98 | 50 | (GA)_15_ | 1 | 1 |
| mPgCIR 99 | 55 | (GA)_20_/(GAT)_14_ | 2 | 1 |
| mPgCIR 100 | 50 | (GA)_15_/(GGAA)_3_ | 3 | 2 |
| mPgCIR 104 | 50 | (GGTT)_3_ | 3 | 1 |
| mPgCIR 105 | 50 | (GA)_30_/(GAAAGA)_3_ | 1 | 1 |
| mPgCIR 108 | 50 | (GA)_13_/(GGAG)_3_ | 1 | 1 |
| mPgCIR 111 | 50 | (GA)_19_ | 1 | 1 |
| mPgCIR 125 | 50 | (GT)_13_/(GTGA)_3_ | 1 | 1 |
| mPgCIR 127 | 55 | (GGTT)_3_/(TATA)_3_ | 1 | 1 |
| mPgCIR 128 | 50 | (GT)_8_/(ATTT)_3_ | 3 | 2 |
| mPgCIR 130 | 50 | (GT)_8_/(GA)_6_ | 2 | 1 |
| mPgCIR 135 | 50 | (GA)_12_ | 2 | 1 |
| mPgCIR 137 | 55 | (GA)_18_ | 1 | 1 |
| mPgCIR 139 | 54 | (GT)_9_/(GA)_9_ | 1 | 1 |
| mPgCIR 144 | 50 | (GA)_17_ | 1 | 1 |
| mPgCIR 148 | 50 | (GA)_19_/(GGGT)_3_ | 1 | 1 |
| mPgCIR 153 | 50 | (GA)_20_ | 1 | 1 |
| Supplement 1 – Continue | | | | |
| **SSR locus** | **T_A_** | **Motif** | **Allele number for each SSR primer in *P.guineense*** | **Allele number for each SSR primer in *P. guajava*** |
| mPgCIR 155 | 50 | (CGTA)_3_/(AAAGT)_3_ | 1 | 1 |
| mPgCIR 157 | 50 | (GA)21/(GGAA)3 | 2 | 1 |
| mPgCIR 158 | 50 | (GT)_13_ | 1 | 1 |
| mPgCIR 160 | 50 | (GA)_10_ | 2 | 1 |
| mPgCIR 161 | 55 | (GA)_15_/(GA)_6_ | 1 | 1 |
| mPgCIR 162 | 50 | (GA)_9_/(GCGC)_3_ | 1 | 1 |
| mPgCIR 167 | 50 | (CGTA)_3_ | 1 | 1 |
| mPgCIR 171 | 50 | (GA)_31_ | 1 | 1 |
| mPgCIR 173 | 56 | (GA)_24_ /(GA)_6_ | 1 | 1 |
| mPgCIR 174 | 56 | (GA)_20_/(GA)_9_/(GGTC)_3_ | 1 | 1 |
| mPgCIR 175 | 56 | (GA)_16_ | 1 | 1 |
| mPgCIR 179 | 54 | (GA)_16_ | 1 | 1 |
| mPgCIR 184 | 55 | (GA)_23_/(GAGG)_3_ | 1 | 1 |
| mPgCIR 186 | 55 | (GA)_18_/(GA)_8_/(GA)_15_ | 2 | 2 |
| mPgCIR 187 | 55 | (GGAA)_3_ | 1 | 1 |
| mPgCIR 188 | 55 | (GA)_9_ | 1 | 1 |
| mPgCIR 192 | 50 | (GA)_23_ | 1 | 2 |
| mPgCIR 198 | 55 | (GA)_22_/(GGTTGA)_3_ | 2 | 2 |
| mPgCIR 200 | 55 | (GA)_6_ | 1 | 1 |
| mPgCIR 202 | 50 | (GA)_17_/(GAAA)/(GA)_6_/(GCGA)_5_ | 1 | 1 |
| mPgCIR 206 | 55 | (GA)_8_/(GT)_11_ | 1 | 1 |
| mPgCIR 208 | 55 | (GA)_29_/(GAG)_5_ | 1 | 2 |
| mPgCIR 209 | 55 | (GA)_15_ | 1 | 2 |
| mPgCIR 211 | 55 | (GT)_8_ | 1 | 1 |
| mPgCIR 212 | 55 | (GA)_23_ | 1 | 2 |
| mPgCIR 214 | 55 | (GT)_6_ | 1 | 1 |
| mPgCIR 216 | 55 | (CGTA)_3_ | 1 | 1 |
| mPgCIR 220 | 55 | (GT)_8_/(GA)_20_ | 3 | 1 |
| mPgCIR 225 | 55 | (GT)_9_/(AGAA)_3_ | 1 | 1 |
| mPgCIR 226 | 50 | (CGTA)_3_ | 2 | 1 |
| mPgCIR 231 | 55 | (GT)_9_/(GA)_12_ | 1 | 2 |
| mPgCIR 233 | 50 | (GA)_22_ | 1 | 1 |
| mPgCIR 234 | 50 | (GT)_11_ | 1 | 1 |
| mPgCIR 235 | 55 | (GA)_28_/(GAGT)_4_ | 1 | 2 |
| mPgCIR 237 | 55 | (GA)_16_ | 1 | 1 |
| mPgCIR 238 | 55 | (GT)_9_/(GT)_8_ | 1 | 1 |
| mPgCIR 242 | 55 | (GA)_12_ | 3 | 2 |
| mPgCIR 243 | 55 | (GA)_29_ | 2 | 1 |
| mPgCIR 245 | 55 | (GA)_11_(GGTA)_3_ | 1 | 1 |
| mPgCIR 247 | 55 | (GA)_20_ | 1 | 1 |
| mPgCIR 253 | 55 | (GA)_29_ | 2 | 2 |
| mPgCIR 254 | 50 | (GT)_16_ | 2 | 1 |
| mPgCIR 255 | 50 | (GA)_27_/(GA)_23_ | 3 | 1 |
| mPgCIR 256 | 50 | (GA)_12_ | 4 | 2 |
| mPgCIR 271 | 55 | (GT)_8_(TGAG)_3_ | 1 | 1 |
| mPgCIR 272 | 50 | (ACAGG)_4_(GACTGG)6 | 2 | 2 |
| mPgCIR 277 | 50 | (TG)_11_(GT)_9_ | 2 | 2 |
| Supplement 1 – Continue | | | | |
| **SSR locus** | **T_A_** | **Motif** | **Allele number for each SSR primer in *P.guineense*** | **Allele number for each SSR primer in *P. guajava*** |
| mPgCIR 287 | 50 | (AG)_35_(TG)_8_ | 1 | 1 |
| mPgCIR 284 | 55 | (AC)_10_ | 2 | 1 |
| mPgCIR 285 | 50 | (GA)_11_(GA)_18_ | 1 | 1 |
| mPgCIR 288 | 50 | (CT)_19_ | 1 | 1 |
| mPgCIR 290 | 50 | (AG)_23_ | 2 | 1 |
| mPgCIR 316 | 50 | (GA)_24_ | 3 | 3 |
| mPgCIR 326 | 50 | (CT)_16_ | 1 | 1 |
| mPgCIR 334 | 55 | (AG)_25_ | 2 | 3 |
| mPgCIR 347 | 55 | (GA)_6_(GA)_13_ | 1 | 1 |
| mPgCIR 374 | 50 | (TC)_19_(CA)_10_ | 1 | 1 |
| mPgCIR 378 | 55 | (CT)_10_(CT)_6_ | 1 | 2 |
| mPgCIR 389 | 55 | (TG)_6_ (GT)_11_ (TC)_10_ | 2 | 2 |
| mPgCIR 392 | 55 | (AC)_10_ | 1 | 2 |
| mPgCIR 399 | 55 | (TC)_7_(TC)_11_(TC)_10_(CA)_14_(CA)_8_ | 1 | 1 |
| mPgCIR 405 | 50 | (GA)_32_ | 1 | 1 |
| mPgCIR 416 | 55 | (TG)_8_(AG)_6_(GAGG)_3_ | 2 | 1 |
| mPgCIR 419 | 55 | (CT)_17_ | 1 | 1 |
| mPgCIR 420 | 55 | (CA)_15_ | 3 | 1 |
| mPgCIR 422 | 55 | (GA)_10_ | 3 | 3 |
| mPgCIR 426 | 55 | (CT)_17_ | 1 | 1 |
| mPgCIR 432 | 55 | (GA)_34_(GTGTC)_4_ | 1 | 1 |
| mPgCIR 437 | 55 | (AC)_10_ | 1 | 1 |
| mPgCIR 439 | 55 | (CA)_8_(ACAC)_4_ | 2 | 1 |
